# Emerging links between homeostatic synaptic plasticity and neurological disease

**DOI:** 10.3389/fncel.2013.00223

**Published:** 2013-11-21

**Authors:** Joyce Wondolowski, Dion Dickman

**Affiliations:** Department of Biology, University of Southern CaliforniaLos Angeles, CA, USA

**Keywords:** synaptic plasticity, homeostasis, neurological disease, retrograde signaling, presynaptic plasticity

## Abstract

Homeostatic signaling systems are ubiquitous forms of biological regulation, having been studied for hundreds of years in the context of diverse physiological processes including body temperature and osmotic balance. However, only recently has this concept been brought to the study of excitatory and inhibitory electrical activity that the nervous system uses to establish and maintain stable communication. Synapses are a primary target of neuronal regulation with a variety of studies over the past 15 years demonstrating that these cellular junctions are under bidirectional homeostatic control. Recent work from an array of diverse systems and approaches has revealed exciting new links between homeostatic synaptic plasticity and a variety of seemingly disparate neurological and psychiatric diseases. These include autism spectrum disorders, intellectual disabilities, schizophrenia, and Fragile X Syndrome. Although the molecular mechanisms through which defective homeostatic signaling may lead to disease pathogenesis remain unclear, rapid progress is likely to be made in the coming years using a powerful combination of genetic, imaging, electrophysiological, and next generation sequencing approaches. Importantly, understanding homeostatic synaptic plasticity at a cellular and molecular level may lead to developments in new therapeutic innovations to treat these diseases. In this review we will examine recent studies that demonstrate homeostatic control of postsynaptic protein translation, retrograde signaling, and presynaptic function that may contribute to the etiology of complex neurological and psychiatric diseases.

## Introduction

Constraining nervous system activity within stable physiological ranges is critical for robust and reliable brain function. However, this stability must also permit the flexibility necessary for learning and memory to occur during the life experiences of an organism. While various forms of Hebbian plasticity have been shown to potentiate or weaken individual synaptic strengths, these mechanisms are inherently destabilizing and would lead to unconstrained activity if left unchecked. Homeostatic processes have therefore been postulated to counteract the instability generated through Hebbian forces, adjusting synaptic strengths and intrinsic neuronal excitability to keep neural circuits functioning within stable dynamic ranges throughout developmental, experiential, and environmental challenges. While homeostatic plasticity is fundamental and conserved in the nervous systems of invertebrates, mammals, and humans, our understanding of the underlying mechanisms of these complex and robust signaling systems has been quite limited.

In the late 1990s, Gina Turrigiano and colleagues reported robust homeostatic synaptic plasticity in cultured rodent neurons (Turrigiano et al., 1998). Around this same time, investigations in *Drosophila* of postsynaptic receptor mutants at the neuromuscular junction (NMJ) also revealed robust homeostatic control of synaptic strength (Petersen et al., 1997; Davis and Goodman, 1998b). Many groups have since described homeostatic adaptations in diverse systems and organisms. Some of these homeostatic processes are thought to require retrograde signaling processes and presynaptic expression (Davis, 2006) while others appear to be postsynaptically induced and expressed (Turrigiano, 2008; Pozo and Goda, 2010).

Beyond conceptual ideas that disruptions in homeostatic synaptic plasticity could lead, in principle, to neural excitability disorders like epilepsy, compelling links with disease had remained elusive. Although synaptic homeostasis has been demonstrated to be a fundamental signaling system observed in a variety of diverse organisms including crustaceans, *C. elegans*, *Drosophila melanogaster*, rodents, and humans (Grunwald et al., 2004; Turrigiano and Nelson, 2004; Marder and Goaillard, 2006; Pozo and Goda, 2010; Turrigiano, 2012; Vitureira et al., 2012; Frank, 2013), direct associations with disease were unclear. Of course, this was probably due in no small part to our poor understanding, particularly on a molecular and cellular level, of both homeostatic synaptic plasticity and neurological and psychiatric diseases. However, work over the past 5 years has revealed exciting new insights into both of these processes, which in turn has led to the discovery of tantalizing links between diseases of the nervous system and synaptic homeostasis. Although just a beginning, a strong conceptual framework has now been established as a foundation to investigate the extent to which defects in homeostatic synaptic signaling could plausibly contribute to the disease pathogenesis of an array of neuropsychiatric and neurological conditions.

**Figure 1:**
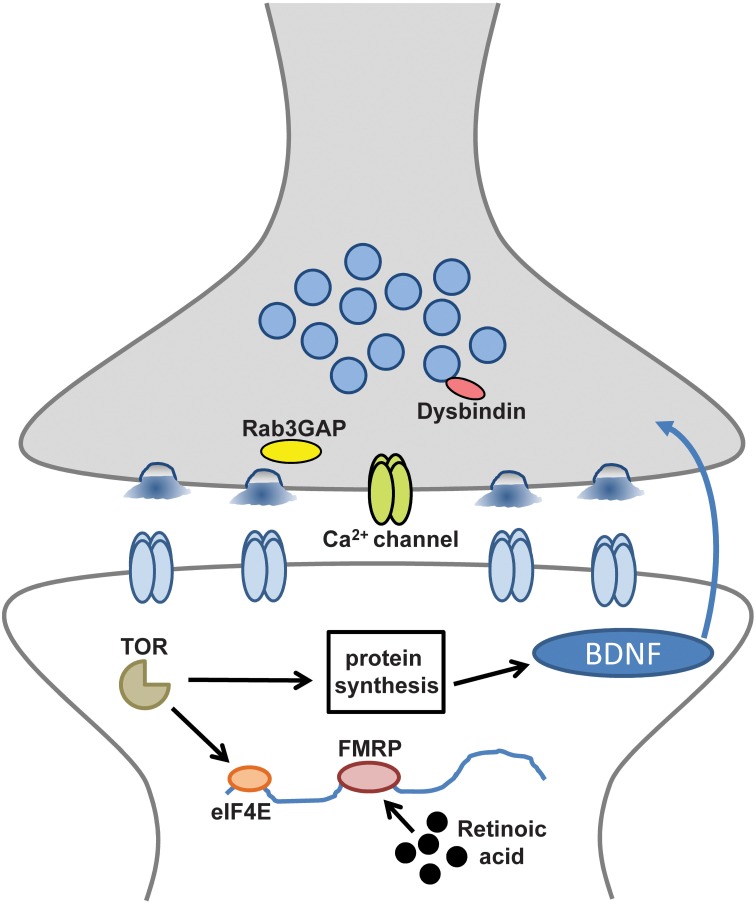
**Disease-related molecules and pathways required for retrograde and presynaptic homeostatic signaling**. Synaptic components and processes involved in retrograde signaling and presynaptic homeostatic plasticity that may also contribute to neurological and neuropsychiatric diseases.

These intriguing links include disease susceptibility genes on both sides of the synapse that appear to help orchestrate the homeostatic control of synaptic function (Table 1) (Pozo and Goda, 2010; Wang et al., 2011a; Yizhar et al., 2011; Qiu et al., 2012). Several theories have been proposed to explain the general mechanisms of neural or synaptic dysfunction that might underlie these disorders (Kehrer et al., 2008; Sudhof, 2008; Yizhar et al., 2011). Disruption or dysregulation of homeostatic synaptic plasticity could be one cause of the excitation/inhibition imbalances that have been recently implicated in cognitive and developmental deficits of the nervous system (Kehrer et al., 2008; Rubenstein, 2010). Indeed, it is tempting to speculate that the high rate of seizures linked with many neurological and neuropsychiatric diseases (Lhatoo and Sander, 2001; Leung and Ring, 2013) could be explained, in part, by defects in homeostatic plasticity. Although compelling studies suggest many of these processes target postsynaptic receptor trafficking and synaptic scaling (Table 1), given the topical focus of this FCN issue, this review will focus on the disease-related pathways linked to the retrograde and presynaptic control of homeostatic plasticity (Figure 1).

**Table 1:** **Genes and molecules required for homeostatic synaptic plasticity and linked with neurological diseases**.

**Molecule**	**Disease**	**Process**	**References**
Arc/Arg3.1	Angelman Syndrome	Required for homeostatic control of postsynaptic AMPA receptor trafficking in mammalian *in vivo* and *in vitro* studies.	Gao et al., 2010; Beique et al., 2011; Cao et al., 2013; Korb et al., 2013
BDNF	Autism, depression, schizophrenia, neurodegeneration, others.	Demonstrated to act as a retrograde messenger in response to AMPA receptor blockade to induce homeostatic presynaptic potentiation in hippocampal neuronal cultures.	Jakawich et al., 2010
Ca^2+^ Channels	Migraine, ataxia, epilepsy, autism, ADHD, bipolar disorder, depression, and schizophrenia	Required for the expression of presynaptic homeostatic plasticity at the *Drosophila* NMJ and cultured mammalian neurons.	Terwindt et al., 1998; Cao and Tsien, 2005; Frank et al., 2006; Zhao et al., 2011; Smoller et al., 2013
Dysbindin	Schizophrenia	Required presynaptically for the homeostatic increase in synaptic vesicle release at the *Drosophila* NMJ.	Dickman and Davis, 2009; Zuo et al., 2009
FMRP	Fragile X Syndrome, autism, intellectual disability	Necessary for the expression of RA-mediated homeostatic increases in AMPA receptor expression and decreases in GABA receptor expression in rodent hippocampal cultures.	Soden and Chen, 2010; Sarti et al., 2013
MeCP2	Rett Syndrome (autism)	Necessary for cell-autonomous homeostatic synaptic scaling up and increases in cellular excitability in response to reduced circuit activity *in vitro*, sensory deprivation *in vivo* in mouse visual cortex, and for synaptic down-scaling in mouse hippocampal culture.	Blackman et al., 2012; Qiu et al., 2012; Zhong et al., 2012; Sala and Pizzorusso, 2013
mTOR/eIF4E	Tuberous Sclerosis Complex (TCS)	Required postsynaptically for retrograde regulation of presynaptic homeostatic potentiation at the *Drosophila* NMJ and in mammalian neuronal cultures.	Penney et al., 2012; Talos et al., 2012
Acetylcholine receptors (AChRs)	Myasthenia Gravis	Loss of postsynaptic AChRs leads to presynaptic homeostatic compensation in quantal content in human muscle biopsies and mouse models.	Plomp et al., 1992, 1995
Narp	Epilepsy	Narp is necessary for homeostatic increases in interneuron excitatory synapses in response to increased network activity.	Chang et al., 2010
Neurexin Neuroligin	Autism, schizophrenia, Tourette's Syndrome	Important for presynaptic homeostatic increases in quantal content at the mouse NMJ.	Sons et al., 2006; Sudhof, 2008
NMDA Receptors/eEF2	Depression	Blocking NMDA receptors may prevent eEF2 phosphorylation, increasing translation, and possibly leading to postsynaptic up-scaling of AMPA receptors.	Kavalali and Monteggia, 2012
Rab3GAP	Warburg Micro and Martsolf Syndrome	Required presynaptically for the induction and expression of homeostatic increases in release probability at the fly NMJ.	Aligianis et al., 2006; Muller et al., 2011

A summary genes and molecules associated with neurological diseases in a variety of systems that have been implicated in pre- and postsynaptic signaling mechanisms driving the homeostatic control of synaptic strength.

## Diseases associated with postsynaptic modulation of protein synthesis and homeostatic retrograde signaling

Several research groups have revealed a complex homeostatic feedback system in the postsynaptic cell that serves to initiate and convey retrograde signaling information back to the presynaptic neuron. These signaling molecules include retinoic acid (RA) (Wang et al., 2011b), glial-derived TNF-alpha (Stellwagen and Malenka, 2006), endocannabinoids (Zhang et al., 2009; Kim and Alger, 2010), and brain-derived neurotrophic factor (BDNF) (Jakawich et al., 2010; Henry et al., 2012). Both RA and BDNF have been implicated in a variety of biological processes and diseases, and multiple lines of evidence suggest that perturbations to postsynaptic glutamate receptors lead to RA and BDNF synthesis in the postsynaptic cell and subsequent alteration of synaptic protein synthesis and retrograde signaling that homeostatically modulate presynaptic function (Jakawich et al., 2010; Chen et al., 2012; Henry et al., 2012; Kavalali and Monteggia, 2012). Interestingly, in the case of several neuropsychiatric diseases including Tuberous Sclerosis Complex and Fragile X Syndrome, imbalances in postsynaptic protein synthesis have been implicated in their etiology. These neuropsychiatric diseases are associated with intellectual disability and autism spectrum disorders, and may arise due to synaptic pathophysiology because of imbalances in the control of postsynaptic protein synthesis (Auerbach et al., 2011; Martin and Huntsman, 2012). Hence, a core postsynaptic step in homeostatic signaling, control of local protein synthesis, may contribute to retrograde signaling and the synaptic dysfunction contributing to these diseases.

### Tuberous sclerosis complex (TSC)

Tuberous Sclerosis Complex is a genetic disease characterized by formation of non-malignant tumors in the brain and other organs (Osborne et al., 1991; Curatolo et al., 2008). This disease is associated with seizures, intellectual disability, and autism, and is the result of heterozygous mutations in the genes encoding *TSC1* or *TSC2*, which encode proteins that together form a complex that regulates activity of the mammalian Target of Rapamycin (mTOR), a well-known regulator of mRNA translation (Zeng et al., 2008; Curatolo and Moavero, 2012; Ryther and Wong, 2012; Talos et al., 2012). mTOR is a constituent of a complex that modulates protein translation by interacting with eIF4E and other translation control factors, suggesting that aberrant regulation of synaptic protein synthesis may contribute to the neurological diseases associated with TSC.

Recent evidence from two established experimental models for homeostatic synaptic plasticity has revealed that a postsynaptic signaling system, which regulates protein expression via the mTOR pathway, is necessary for the retrograde control of presynaptic release. Using rodent hippocampal neurons in culture, researchers have found that disruption of NMDA receptor mediated miniature postsynaptic currents leads to a rapid increase in postsynaptic protein synthesis and a compensatory increase in postsynaptic glutamate receptor expression (Sutton et al., 2006). Further, alterations in synaptic protein synthesis via eIF4E and mTOR were found to be rapidly induced (within 1–2 h) following acute pharmacological blockade of AMPA receptor function using CNQX (Jakawich et al., 2010; Henry et al., 2012). Interestingly, one substrate of this postsynaptic signaling system is BDNF, which is synthesized and released postsynaptically where it activates presynaptic TrkA receptors and enhances presynaptic function (Jakawich et al., 2010). Dysfunction in this acute, retrograde modulation of presynaptic function via postsynaptic protein synthesis could plausibly contribute to the social, cognitive, and excitability disorders observed in TSC and other neurological diseases, including epilepsy.

This same pathway that modulates postsynaptic protein synthesis and leads to the retrograde enhancement of presynaptic transmission via the eIF4E and the TOR translational control factors has also been demonstrated in a separate model system for presynaptic homeostatic plasticity, the *Drosophila* NMJ (Penney et al., 2012). In this system, pharmacological or genetic perturbations to postsynaptic glutamate receptor function, akin to the protocols used in rodent preparations discussed above, lead to a currently unknown retrograde signal the potentiates presynaptic release (Davis and Goodman, 1998b; Davis, 2006; Frank, 2013). Penney and colleagues found that when levels of postsynaptic eIF4E or TOR proteins were reduced, this led to a disruption in the compensatory homeostatic increase in presynaptic release normally observed following reduction of postsynaptic glutamate receptor function (Penney et al., 2012). Although there is no obvious fly counterpart of BDNF and the identity of the retromer is unknown, it appears that fundamental, evolutionarily conserved parallels between Drosophila and rodents in postsynaptic signaling via eIF4E and TOR can modulate the retrograde, homeostatic control of presynaptic release. This fundamental property of synaptic homeostasis may be dysregulated during TSC. Interestingly, expression of another protein, neurexin, is controlled by eIF4E (Gkogkas et al., 2013), and has been linked with autism spectrum disorders (Sudhof, 2008), providing another putative link between homeostatic regulation of the synapse and neuropsychiatric disorders.

### Fragile X syndrome (FXS)

Fragile X Syndrome is a neuropsychiatric disorder characterized by intellectual disability, deficits in social interaction, and associated neurological conditions such as seizures (Bagni and Greenough, 2005; Bhakar et al., 2012). This genetic syndrome is the most widespread single-gene cause of autism and inherited form of mental retardation in boys (Hagerman and Hagerman, 2002; Hernandez et al., 2009). FXS is the result of mutations in the fragile X mental retardation gene (*fmr1*), which encodes the Fragile X Mental Retardation Protein (FMRP), and is necessary for normal neural development (Lhatoo et al., 2001b). Research over the past ten years has demonstrated that FMRP is a synaptic RNA binding protein that represses translation of a subset of RNAs; hundreds of FMRP targets have recently been discovered (Bassell and Warren, 2008; Costa-Mattioli et al., 2009; Zukin et al., 2009; Darnell et al., 2011), leading to models in which both pre- and post-synaptic RNAs are under modulatory translational control by FMRP (Feng et al., 1997; Stefani et al., 2004; Christie et al., 2009; Akins et al., 2012).

The intercellular signaling molecule RA has been found to regulate homeostatic synaptic plasticity in rodent neuronal cultures (Aoto et al., 2008; Maghsoodi et al., 2008; Soden and Chen, 2010; Chen et al., 2012). Here, RA was demonstrated to be required for the homeostatic increase in synaptic strength following postsynaptic receptor perturbation (Aoto et al., 2008). Specifically, blockade of excitatory activity was induced by TTX and APV treatment, and RA was necessary for homeostatic postsynaptic scaling through AMPA receptor insertion (Chen et al., 2012). A novel cytosolic and synaptic function for RA was determined, as synthesis of RA was induced following this homeostatic challenge. RA binds to its receptor RARalpha and in turn modulates postsynaptic protein expression (Lane and Bailey, 2005; Chen et al., 2012). Importantly, in addition to the function of RA in regulating postsynaptic homeostatic glutamate receptor scaling, RA was also observed to have roles in modulating presynaptic function, as reflected in increased mEPSC frequency (Wang et al., 2011b; Chen et al., 2012). Intriguingly, FMRP is also necessary for RA-mediated homeostatic plasticity at both excitatory and inhibitory synapses (Soden and Chen, 2010; Sarti et al., 2013). In neurons isolated from *fmr1* mutant mice, bi-directional homeostatic scaling was not observed, while other forms of plasticity remained intact. These findings underscore the importance of tightly regulating synaptic protein synthesis in the context of homeostatic plasticity and the possible neural diseases that may result from dysfunction in this process.

The research described above provides an intriguing body of evidence linking postsynaptic protein synthesis and retrograde signals to both homeostatic synaptic plasticity and neurological diseases. Several diseases show significant overlap in the signaling pathways relevant to trans-synaptic homeostatic signaling. Interestingly, mTOR activity has been implicated in TSC, neurofibromatosis-1, FXS, and a variety of PTEN-associated conditions (Gipson and Johnston, 2012). Regulation of postsynaptic protein synthesis via mTOR and FMRP have both been shown to disrupt mGluR dependent LTD (Auerbach et al., 2011). Indeed, there are even links between FXS and BDNF signaling (Leung and Ring, 2013). The question of how similar signaling systems underlying homeostatic and Hebbian (LTD/LTP) mechanisms are integrated to ensure flexible yet stable synaptic function remains an important area for future research.

Emerging evidence suggests that problems with retrograde signaling may be involved in an array of autism spectrum disorders beyond FXS and TSC. Another example of trans-synaptic signaling being linked to cognitive disease is seen with neurexins and neuroligins, cell-adhesion molecules that are known to regulate synaptic function (Sudhof, 2008). These molecules have been linked with a variety of diseases including autism (Yan et al., 2005; Szatmari et al., 2007; Kim et al., 2008), schizophrenia (Kirov et al., 2008; Walsh et al., 2008), and other neuropsychiatric conditions (Lawson-Yuen et al., 2008; Sudhof, 2008). Intriguingly, neurexins have been found to be necessary for presynaptic homeostatic plasticity at the mammalian NMJ (Sons et al., 2006), providing another possible link between homeostatic plasticity and neuropsychiatric disease.

## Diseases associated with presynaptic mechanisms of homeostatic plasticity

### Calcium channel and homeostatic signaling in migraine and epileptic diseases

Migraine is the most common neurological disease, thought to affect 10–15% of the general population (Goadsby et al., 2002; Wessman et al., 2007). Insight into the etiology of these often debilitating headaches may be uncovered through the study of familial hemiplegic migraine type 1 (FHM1), a rare, inherited form of the disease (Ophoff et al., 1996). FHM1 is caused by missense mutations in P/Q type calcium channels, which are involved in neurotransmitter release and presynaptic function (Zhang et al., 1993; Lhatoo et al., 2001a). These mutations cause impairments in the ability of these channels to conduct Ca^2+^ currents triggered by action potentials (Kraus et al., 1998; Hans et al., 1999; Cao et al., 2004). P/Q-type calcium channels have fundamental roles in mediating fast synaptic transmission at nerve terminals. Autosomal dominant mutations in the CACNA1A gene, which encodes the voltage-gated P/Q-type calcium channel alpha1 subunit, has also been associated with cerebellar ataxia, vertigo, epilepsy, and schizophrenia (see below) in addition to FHM (Lhatoo et al., 2001a). CACNA1 and CACNB2 channelopathies have also been implicated in several other diseases, including autism, attention-deficit hyperactivity disorder, bipolar disorder, and major depressive disorder, indicating that calcium channel signaling may have crucial functions that, when perturbed, may contribute to psychopathological susceptibility (Terwindt et al., 1998; Lee et al., 2013; Smoller et al., 2013). These studies also suggest a disruption in the balance of excitatory and inhibitory activity that may ultimately lead to the pathogenic states of these diseases.

Interestingly, mutations in the *Drosophila* P/Q calcium channel *cacophony* (*cac*) have been found to disrupt homeostatic plasticity at the fly NMJ (Frank et al., 2006, 2009). Hypomorphic mutations in *cac* lead to reduced presynaptic calcium influx and prevent the retrograde, homeostatic increase in presynaptic release. Recent studies using calcium imaging techniques demonstrate that an acute increase in presynaptic calcium influx through Cac is necessary for the expression of homeostatic plasticity (Muller and Davis, 2012; Younger et al., 2013). In addition, regulation of presynaptic calcium influx is also required for homeostatic synaptic plasticity in the mammalian hippocampus (Zhao et al., 2011). Since calcium signaling is known to play a critical role in many aspects of neurotransmission, the function of these channels in neurological disease is certainly not limited to homeostatic plasticity. This topic is covered in more detail in a forthcoming review (Frank, *in review*).

### Myasthenia gravis (MG)

Myasthenia gravis is an autoimmune disease that causes muscle weakness and fatigue. In a majority of cases, a loss of postsynaptic acetylcholine (ACh) receptors is observed at the NMJ due to autoantibody production targeting these receptors. In recordings from muscles obtained from MG patients and also from toxin-induced animal models of the disease, miniature end-plate potentials (mEPPs) show dramatically reduced amplitudes (Plomp et al., 1992, 1995). However, evoked end-plate potentials (EPPs) are not significantly reduced due to increased quantal content, indicating that homeostatic processes at the human NMJ, like that of the fly NMJ, compensate for this perturbation of postsynaptic receptor function (Cull-Candy et al., 1980; Plomp et al., 1992, 1995; Davis and Goodman, 1998a). This homeostatic increase in ACh release could compensate for the reduced postsynaptic excitability of the muscle. However, if the compensation is not sufficient to restore all EPPs to suprathreshold activation, then defects in muscle activity would still be observed. Indeed, in a subset of recordings mEPP amplitudes were below the detection limit and showed smaller than normal EPPs, likely indicating limitations in homeostatic compensation at these synapses (Plomp et al., 1995). Furthermore, the increased release probability likely contributes to increased short-term depression, leading to rapid exhaustion of neurotransmitter vesicles and rapid-onset muscle fatigue. Ultimately, disruption of postsynaptic receptor function supersedes the homeostatic processes and major disease progression ensues.

A subclass of MG is caused by production of antibodies that attack muscle specific kinase (MuSK) (Hoch et al., 2001; Viegas et al., 2012), a postsynaptic receptor tyrosine kinase known to be important in receptor clustering during NMJ synaptic development and maintenance (DeChiara et al., 1996; Sanes and Lichtman, 2001; Kong et al., 2004). In recordings from muscles obtained from MuSK MG patients (Niks et al., 2010) and from mice immunized against MuSK (Viegas et al., 2012), mEPPs were found to be significantly reduced. However, in contrast to the NMJ studies in mice and human discussed above, there was no evidence of increased presynaptic release in MuSK-dependent MG, indicating a lack of homeostatic expression (Niks et al., 2010; Viegas et al., 2012). Compared to AChR-associated MG, MuSK-related MG is more severe, is treatment-resistant, and is observed more broadly in the musculature, affecting bulbar, facial, and respiratory muscles as well (Evoli et al., 2003), which is likely due, in part, to a lack of homeostatic compensation. These experiments suggest that MuSK may be required as part of the postsynaptic homeostatic signaling system that initiates a retrograde, presynaptic change in release.

### Warburg micro and martsolf syndrome

Rab3 proteins are known to regulate vesicular membrane transport and play a key role in modulating calcium-dependent neurotransmitter release (Sakane et al., 2008). The activity of Rab3 is regulated in part by Rab3 GTPase-activating protein (Rab3GAP), which converts active Rab3-GTP to in the inactive GDP-bound form. Rab3GAP is a heterodimeric protein composed of catalytic and non-catalytic subunits, mutations of which cause Warburg Micro syndrome and Martsolf syndrome (Aligianis et al., 2006). Warburg Micro syndrome is an autosomal recessive disorder that causes severe intellectual disability, ocular defects, and microcephaly (Dursun et al., 2012). Martsolf syndrome shares many clinical features with Warburg Micro syndrome, but is less severe and is associated with milder cognitive dysfunction (Aligianis et al., 2006). Recent evidence suggests that Rab3GAP is critically involved in the induction and long-term maintenance of homeostatic synaptic plasticity. Drosophila Rab3GAP mutants do not show homeostatic increases in neurotransmitter release at the NMJ in response to pharmacological or genetic disruption of postsynaptic glutamate receptors (Muller et al., 2011). Rab3GAP appears to work through Rab3 GTPase to negatively regulate the expression of presynaptic homeostatic plasticity. Thus, the disruption of homeostatic synaptic plasticity may contribute to the cognitive and developmental defects associated with these Warburg Micro and Martsolf syndromes.

### Schizophrenia

Calcium signaling, through the implication of CACNA1, has been implicated in several brain disorders including schizophrenia, and a fly calcium channel, Cac, has been found to block homeostatic synaptic plasticity (see above). In addition to these findings, genetic screens in *Drosophila* have revealed unanticipated links with schizophrenia. As alluded to above, the *Drosophila* NMJ has been established as a model system to characterize homeostatic synaptic plasticity. Pharmacological or genetic manipulations that reduce postsynaptic receptor function lead to a compensatory increase in presynaptic release that precisely offsets the perturbation and restores normal postsynaptic excitability (Davis and Goodman, 1998b; Davis, 2006; Frank, 2013). Given the rich genetic resources available in flies, electrophysiology-based forward genetic screens have recently been pioneered to identify genes, that when mutated, lead to defective homeostatic synaptic plasticity (Dickman and Davis, 2009; Muller et al., 2011).

One of the first genes identified from these screening efforts was the *Drosophila* homolog of the vertebrate gene *dysbindin* (Dickman and Davis, 2009). Interestingly, the human homolog of this gene, *DTNBP1*, has been identified as a primary schizophrenia susceptibility gene in a variety of Genome Wide Association Studies (Ross et al., 2006; Zuo et al., 2009; Ghiani and Dell'Angelica, 2011). In Drosophila, Dysbindin was found to be required presynaptically for both the acute induction and long term expression of synaptic homeostasis (Dickman and Davis, 2009). Dysbindin localized to presynaptic vesicles and was demonstrated to alter the calcium dependence of synaptic vesicle release in a unique way, where mutants exhibited severely reduced baseline release, but only at low external calcium concentrations, whereas overexpression of *dysbindin* led to a potentiation of baseline release, but only at higher external calcium levels (Dickman and Davis, 2009). Subsequent work found that Snapin, a synaptic vesicle protein that binds to Dysbindin, was also required for homeostatic synaptic plasticity and was necessary for the potentiation of baseline synaptic transmission by Dysbindin (Dickman et al., 2012). In rodent studies, *dysbindin* was found to be necessary for proper synaptic glutamate release and neurotransmission (Numakawa et al., 2004; Chen et al., 2008), while mouse mutants even displayed behavioral deficits akin to schizophrenia-like conditions (Chen et al., 2008). It is thus interesting to speculate that Dysbindin may homeostatically tune synaptic strength, and deficits in this process may give rise to cognitive and behavioral conditions of relevance to the etiology of schizophrenia.

Dysbindin and Snapin are part of a larger protein complex called the Biogenesis of Lysosome-related Organelles Complex-1 (BLOC-1), composed of eight total subunits (Ghiani and Dell'Angelica, 2011; Mullin et al., 2011). While the molecular function of the BLOC-1 complex is unclear, it appears to be involved in vesicle trafficking (Ghiani and Dell'Angelica, 2011; Larimore et al., 2011). Consistent with such a role in the nervous system, both Dysbindin and Snapin appear to function in synaptic vesicle trafficking and may be involved in other synaptic membrane trafficking pathways. Interestingly, another component of the BLOC-1 complex, Muted, has recently been associated with schizophrenia (Morris et al., 2008; Ryder and Faundez, 2009). These findings underscore the intriguing possibility that defective synaptic membrane trafficking through components of the BLOC-1 complex in the context of homeostatic synaptic plasticity may contribute to the etiology of complex neuropsychiatric diseases like schizophrenia.

## Concluding remarks

Tantalizing links have recently been uncovered that suggest synaptic processes necessary for the homeostatic control of synaptic function, when defective, may contribute to the pathogenesis of a variety of neurological and neuropsychiatric diseases. Importantly, several intriguing and novel genes and molecules have been revealed to disrupt these complex and adaptive homeostatic signaling systems (Figure 1). The challenge moving forward will be to understand how these individual genes and molecules work together to integrate homeostatic information and orchestrate stable cellular, synaptic, circuit, and systemic functions in the brain. It is tempting to speculate that the etiologies of many seemingly disparate neurological and neuropsychiatric diseases share common dysfunctions in pathways related to homeostatic synaptic plasticity. Indeed, a large genome-wide study sponsored by the National Institute of Mental Health has revealed a high degree of overlap in the genes responsible for five of the most common neuropsychiatric diseases (Lee et al., 2013). A combination of human genetic mapping and patient studies coupled with innovative genetic, electrophysiological, and imaging approaches in model systems are certain to reveal new insights into the processes driving the homeostatic control of synaptic function in health and disease.

### Conflict of interest statement

The authors declare that the research was conducted in the absence of any commercial or financial relationships that could be construed as a potential conflict of interest.
